# The Emetine and Other Treatment of Pyorrhea

**Published:** 1917-08

**Authors:** 


### THE EMETINE AND OTHER TREATMENT OF PYORRHEA

Considerable difference of opinion prevails as to the value of emetine hydrochloride for curing pyorrhea; some dentists condemn it, others are extremely enthusiastic in its praise. The truth probably lies midways: when used in the right way and in the right kind of cases, emetine will effect a cure. A conservative and yet optimistic statement of its place in the therapy of pyorrhea is made by MacDonald in the January number of *The Dental Cosmos* (p. 65), who writes as follows:

"The use of emetine hydrochloride recalls Dr. James Truman's early treatment by packing the pockets with quinine. The similarity of action of the two drugs would account for the successes in his treatment, in view of the work done by Smith and Barrett and by Bass and Johns.

"Emetine has given me such excellent results in several cases that I consider it a very valuable addition to the drugs used in the treatment of pyorrhea. Sometimes it has failed me and sometimes its results have been almost dramatic. Sometimes, injected intramuscularly, it has acted well, and in other cases local use has produced better results. Combined with treatment by ionic medication, it has given me good results. Occasionally the injection into the arm (I

generally inject into the deltoid muscle near the insertion) is followed by some soreness, a small hard lump persisting for some days. In one case, the injection apparently irritated a cutaneous nerve, as a herpetic eruption appeared. In the majority of cases, however, I have not had any disagreeable results. The local soreness may be in great part avoided by kneading the site of the injection, to disperse it more rapidly. I always sterilize the skin before the injection. Do not use emetine in the case of an alcoholic, if you wish to avoid nausea, and so on.

"Another precaution necessary is, to avoid the use of emetine, even locally, during the menstrual period, as it is likely to check the menstrual flow. Just how it acts in that manner is difficult to explain; but, emetine is a drug the therapeutic possibilities of which are not yet thoroughly understood, and I only can refer in this connection to its similar striking results in hemoptysis, as reported by Flandin to the Medical Society of the Paris Hospitals, and published in *Le Monde Médical.*

"I know that many dentists have already abandoned the use of emetine, because they apparently have not obtained the expected results in every case, and their doubt as to the pathogenicity of the ameba has thus seemed to be confirmed. On the other hand, I think Bass and Johns lay too much stress upon the ameba as being alone responsible for pyorrhea. We might cite dysentery as a parallel example in medicine. If all cases of dysentery were treated with emetine alone, the drug would fall into disrepute. The reason for this is that we recognize two forms of dysentery, amebic and bacillary, and the particular form present must be diagnosed before the correct treatment is decided upon.

"May not one form of pyorrhea be due to a pathogenic ameba? I venture the opinion as a result of many successes with emetine. But I emphasize the fact that instrumentation must be a part of the treatment. Occasionally I have obtained good results in an obstinate pyorrheal pocket with a paste of hydrargyri iodidum viride and glycerin packed into the pocket, and then leaving a twist of cotton saturated with glycerin in the pocket for a few hours. The benefit of this treatment I attribute first to the antiseptic power of the drug, combining the action of iodin with that of mercury, and second, to the glycerin inducing an osmotic flushing of the pocket with fresh serum. As the dose is only from 1 to 3 grains, care must be exercised in using hydrargyri iodidum viride.

"Where a pocket persists in spite of all treatment we should be sure that a dead pulp is not adding to our difficulty, or, if in the upper jaw, that the pocket is not connected with the antrum, as in the cases reported by Underwood in the *British Medical Journal*. Immobilization of a loose tooth is helpful if the method used does not involve impingement on the gums or favor lodgment of food débris. All hopeless teeth should be extracted.

"I prefer vaccine treatment where any systemic effects of the pyorrheal condition are in evidence. Here, again, vigorous local measures should be used at the same time, for it seems unreasonable to leave a septic and irritating mass about the teeth and expect good results from a vaccine. I prefer to use a mixed autogenous vaccine. Vaccine treatment and the use of glycerin locally seems to me an ideal method, for we are thus flushing the pockets with fortified serum."—*Clinical Medicine.*

